# Use of telemedicine in obstetrics and gynaecology in Zimbabwe during a lockdown period

**DOI:** 10.11604/pamj.supp.2020.35.2.23675

**Published:** 2020-06-24

**Authors:** Jephat Moyo, Gerald Madziyire

**Affiliations:** 1 Department of Obstetrics and Gynaecology, National University of Science & Technology, Mpilo Hospital, Vera Road, Mzilikazi, Bulawayo, Zimbabwe; 2 Department of Obstetrics and Gynaecology, University of Zimbabwe

**Keywords:** Telemedicine, covid-19, pandemic

## Abstract

**Introduction:**

Telemedicine is an ideal innovation to achieve social distancing, one of the mitigating strategies during the current Covid 19 pandemic. Zimbabwe effected a 21day lockdown from the 2nd of March 2020 to control the spread of the Covid 19 infection. Free teleconsultation service was provided by the researcher. We sought to determine the effectiveness and acceptability of telemedicine in Obstetrics and Gynaecology during this period.

**Methods:**

We conducted a retrospective and prospective analysis of the messages from the WhatsApp consults for demographic characteristics, type and classification of condition, level of urgency, ability to make a diagnosis and the type of recommendation offered. A follow-up message was used to assess effectiveness of the medical advice given and patient satisfaction.

**Results:**

Of 109 women who used telemedicine 67consented. The average age was 31 years and most of the women were married, had university or tertiary college education and were urban dwellers. Forty-nine (73.1%) cases were Gynaecological consults and 51 (76.1%) were elective cases. Twenty (29.9%) and fourteen (20.8%) cases needed elective and urgent hospital referral respectively. A diagnosis was made in 33(49.3%) of the cases from the available information during the consult. Thirty-five (52.2%) cases had recovered whilst 27(40.3%) cases were still waiting further assessments at the end of the follow up. The patients were satisfied with the service in 94% of the cases.

**Conclusion:**

Telemedicine services provided during the lockdown period were effective and acceptable in managing women with Obstetrics and Gynaecological conditions. Telemedicine should be rolled out during this pandemic to limit risk to patients and healthcare providers.

## Introduction

Telemedicine (TM) is the use of electronic information and communication technology to provide direct patient health services when the health provider and patient are separated by physical distance [1]. It can take the form of teleconsultation, tele-monitoring and tele-expertise and in each scenario communication is either between healthcare provider and another health care provider or between patient and a healthcare provider. The communication can be real time or delayed text messaging or voice call, delayed recorded messages or electronic mail [2]. It is well established and rapidly evolving in modern medicine. In Obstetrics and Gynaecology telemedicine is used in prenatal care, maternal and fetomaternal monitoring, fetal echocardiography, monitoring of chronic medical conditions in pregnancy and in reproductive medicine [3]. TM advantages includes reducing office and hospital visits for minor conditions, increasing access to specialist consultation and lowering the cost of healthcare for the patient, health provider and health funders [4,5]. The disadvantages are the elimination of physical examination of the patient, difficult in prescribing tests and medications electronically and sometimes the unwillingness of health care funders to pay for telemedicine consult. The first case of Covid 19 was reported to the World Health Organisation (WHO) on the 31st of December 2019. The highly infectious virus quickly spread to other countries to be declared a global pandemic affecting more than 4 millon people and killing more than 200 000 in 215 countries. Social distancing is one of the mitigating strategies to reduce the spread of Covid 19.

TM is one of the useful innovations that can be used to achieve social distancing by minimizing physical consultations. By using TM, the health worker, vulnerable patient´s groups and non Covid-19 patients are protected from the infection [6]. The need for virtual medical consultation has been accentuated by restrictions to travel within communities imposed by various governments during this pandemic. Zimbabwe in particular effected a three-week lockdown from the 2nd of March 2020. In Zimbabwe TM has not been widely used. No program or study has researched TM in Zimbabwe. Its effectiveness, the demographics of its users, the common presenting conditions, the outcome of the TM consultations and the acceptability of TM by Zimbabwean Obstetrics and Gynaecology patients is unknown. In this project we seek to answer these questions by assessing Obstetrics and Gynaecology consultations made via Short Message Services (SMS) and WhatsApp platform during the lockdown period. Our objectives thus were: 1) To determine the demographic characteristics (mean age, level of education, place of residence and marital status) of women who utilized TM during the lockdown period; 2) To establish the main group of conditions presented; 3) To assess the effectiveness of telemedicine in Obstetrics and Gynaecology 4) To assess patient satisfaction to the TM services provided.

## Methods

When the lockdown was enforced the Zimbabwe Senior Hospital Association (ZHDA) asked for volunteers among its membership who would offer free specialist consulting services to members of the public who could no longer access health facilities due to lockdown measures. The service was authorised by the Medical and Dental Practitioners Council of Zimbabwe (MDPCZ) to start operating during the 21 day lockdown. The service was to run till the restrictions had been relaxed and the pandemic contained. The researcher volunteered to offer consultation in the field of gynaecology and obstetrics. The telephone numbers of the volunteers were advertised on electronic and print media. The objective was to offer medical consultation and triage patients to avoid serious morbidities during a time when movement was severely restricted. There was no need for users to register. The patients would send message via WhatsApp or Short Message Service to the doctor´s number and the doctor would respond in real time or delayed texting. The doctor would then communicate his recommendation to the patient. In cases where the case was deemed an emergency or urgent case the researcher would make arrangements for the patient to be attended in the nearest hospital by contacting Obstetricians and Gynaecologist working in or closest to the facility.

After ethical approval from the Mpilo Institutional Review Board and Medical Research Council of Zimbabwe we conducted a retrospective and prospective study on all consenting Obstetrics and Gynaecology patients who had freely consulted the researcher on WhatsApp platform during the lockdown period as part of social distancing strategy. The retrospective analysis was done by accessing the messages of all consenting patients and checking for completeness and then sending supplementary and follow up questions on the same platform. The prospective patients had their consultations first and then 48hrs later a consent was requested. This was done to limit coercion had the consent been sort first. Universal convenient sampling of all consenting patients who consulted the researcher was done. We evaluated the patient demographics by collecting the age, marital status, level of education and place of residence. The cases were then classified according to whether they were of Gynaecological or Obstetric nature and to their level of urgency. Chronic conditions and/or follow up of previously attended conditions were classified as *Elective.* Those that needed immediate action but were not life threatening were classified as *Urgent* whilst those who needed immediate action and were life threatening were classified as *Emergency.*

The Gynaecological cases were further classified into Infective, Reproductive health disorder, Oncology, Contraceptive and Other cases. The infective cases were those cases whose etiology was of genital tract infections. The reproductive health disorder cases included subfertility cases, gynaecological endocrinology and menstrual disorders. The obstetric cases were classified according to their gestation and booking status. A booked case being defined as a case where the patient had consulted a health practitioner for the pregnancy. The effectiveness of the service was assessed by the ability to reach a clinical conclusion during the consultation from the information gathered only during the consultation and response to the management recommendations which was obtained by a follow-up message. Patient satisfaction was assessed by asking the patient to give a score of 1 to 4, the lowest being very dissatisfied and highest being very satisfied with the services. The collected data was entered into the Statistics Software Epi Info 7 for analysis.

## Results

**Figure 1:**
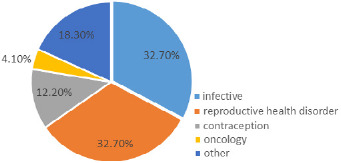
Map of Oyo State highlighting the LGAs of confirmed COVID-19 cases

A total of 109 women consulted the researcher using the WhatsApp platform during the 21day lockdown period and 67 of these consented to be included in the study giving a response rate of 61%. The average age of the women was 31 years with the youngest and the oldest being 19 years and 61years respectively. Most of the women were married (68.7%) and had university or tertiary college education (76%). Fifty-eight (86.5%) were urban dwellers. Three cases were Zimbabweans based in neighboring countries (Table 1). The were 18 obstetric consultations and of these eleven (61.1%) were not booked. The obstetric cases were evenly distributed through the three trimesters. Forty-nine (73.1%) cases were Gynaecological consultations. Of the Gynaecological cases the commonest group of conditions were infective (32.7%) and reproductive health disorders (32.7%) Figure 1. Fifty-one cases (76.1%) were elective whilst three (4.5%) were emergency.

**Table 1:** Demographics and characteristics of the cases

Outcome	Frequency	Percentage	Cumulative percentage
**Marital status**			
Married	46	68.7	68.7
Single	19	28.3	97.0
Divorcee	1	1.5	98.5
Not stated	1	1.5	100
**Level of education**			
High school	6	9	9
Secondary	10	14.9	23.9
Tertiary college	22	32.8	56.7
University	29	43.3	100
**Level of urgency**			
Elective	51	76.1	76.1
Urgent	13	19.4	95.5
Emergency	3	4.5	100
**Obstetric: stage of pregnancy**			
1st trimester	6	33.3	33.3
2nd trimester	5	27.8	61.1
3rd trimester	5	27.8	88.9
postpartum	2	11.1	100

During the consultations the researcher was able to reach a diagnosis or decision using available information in thirty-three (49.3%) of the cases. The researcher needed a diagnostic test or a physical examination in sixteen (23.9%) and eighteen (26.8%) cases respectively. The main recommendation to the patients was an elective referral to a hospital or a specialist. Fourteen cases needed an urgent referral to the hospital for treatment. Thirty-five (52.2%) of these cases reported that they had recovered on follow up whilst twenty seven (40.3%) were still waiting further tests and assessments. Only 5 cases had not recovered despite being given treatment. The majority of the clients were satisfied with the telemedicine consult with 52.2% reporting to be very satisfied. Four (6%) patients were dissatisfied with the service. Three of the cases who were dissatisfied were awaiting assessment for elective conditions. One of the patients was a 19 yrs old who had delivered recently and had tests for Covid 19 and was awaiting the Covid 19 PCR test results (Table 2).

**Table 2:** Effectiveness and outcome

Outcome	frequency	Percentage(%)	Cumulative percentage (%)
**Ability to make diagnosis**			
Able	33	49.3	49.3
Unable diagnostic test needed	16	23.9	73.2
Unable physical examination needed	18	26.8	100
**Recommendations**			
Order a diagnostic test	10	15	15
Re-assure	11	16.4	31.4
Electronic prescription	12	17.9	49.3
Urgent referral to the hospital	14	20.8	70.1
Elective referral to hospital /specialist	20	29.9	100
**Outcome of the intervention**			
Condition resolved	35	52.2	52.2
Awaits further tests and assessment	27	40.3	92.5
Condition unresolved despite the treatment	5	7.5	100
**Patient satisfaction scores**			
Score 4 (very satisfied)	35	52.2	52.2
Score 3 (satisfied)	28	41.8	94
Score 2 (dissatisfied)	4	6	100

## Discussion

The TM service provided during the lockdown period by the researcher was accessed by 109 women in 21 days. These were mainly married, well educated, urban dwelling young women. The demonstrated use by this group of women closely follows the pattern of use and access to internet technology in the population [7]. These are women who are likely to have the electronic communication devices, have access to internet and are tech-savvy. The fewer rural dwellers and less educated women utilizing TM demonstrate the drawbacks, in that equipment, connectivity and literacy plays a role in use of TM. This later group would however benefit more as they have limited access to specialist care. The main cases were elective gynaecological conditions with infective and reproductive health disorders. This is agreement with a study on Polish speaking women which showed that 76% of TM consults in Obstetrics and Gynaecology were gynaecological [8]. The service offered an opportunity to women with chronic conditions to access specialist services and those with follow-up visits affected by the lock down to consult by TM. The high frequency of Infective and reproductive health disorders is in keeping with the prevalence of gynaecological morbidities in our population. Those women with emergency conditions were fewer as the hospitals were still attending to emergency cases.

TM services in this study were effective as the researcher was able to make a confident diagnosis and conditions resolved in 49.3% and in 52.2% of the cases respectively. This is in agreement with findings of a systematic review which showed a benefit of text messaging and remote monitoring in reducing unscheduled hospital visits in pregnancy [9]. TM effectively reduces the number of care seekers who will eventually need physical assessment. Strategies should be put in place to facilitate completion of service for such clients as this study showed that 40.9% of them were delayed by the disruption caused by the lockdown. This study shows that TM can be used effectively to screen patients before they go to a health practitioner or facility. This does not only reduce crowding at health facilities but save resources at public facilities and patients´ time and money and in this pandemic reduce chances of infecting both patients and health workers. The satisfaction scores to the service were very high ranging from satisfied to very satisfied in 94% of the cases. The high score was due to patients having access to free specialist consultation at the convenience of their homes. Pflugeisen BM et al also found that patient satisfaction scores were higher when he compared virtual care to “traditional” obstetric care [10]. The cases of those who were dissatisfied were mainly influenced by the delays caused by the lockdown measures.

TM has been used previously in similar healthy crisis. It has been used in contact tracing, monitoring isolated patients, screening potential infected patients and providing health care to unaffected patients as in our study [11,12]. In a similar crisis in 2015 in South Korea during the MERS-CoV epidemic the Samsung medical center had to be closed due to infection and the government authorized the use of TM to patients who were affected by the closure [13]. This study provides support for rolling out TM as a mode of medical consultation during contagion pandemics. This should be done both in private and public facilities. In the public facilities doctors or nurses can be employed to answer people´s health queries using SMS or WhatsApp service. The service can be designed to direct patients to the practitioner dealing with their area of interest. A fee can be charged to manage the service. Patients would still likely prefer to have professional advice of whether they should go for physical consultation before they incur transportation costs and risk of Covid-19 infection by going to a health facility. In private practice a consultation fee can be levied for the service and medical funders should pay for such consultations. Electronic prescription pads, laboratory and radiology requests should be used. The study was limited by the small sample size. Telemedicine should be rolled out and bigger studies should be done for telemedicine even after pandemic is over to evaluate the role of TM in Gynaecology and Obstetrics practice in the low resource settings. Other interest groups like health funders and policy makers must be involved.

## Conclusion

TM services provided during the lockdown period were effective in diagnosing and treating young married women with mainly elective gynaecological conditions. The majority of conditions resolved resulting in high satisfaction scores for TM from the patients.

### What is known about this topic

- Telemedicine is useful in managing chronic conditions;
- Telemedicine improves monitoring of medical conditions in pregnancy like Blood pressure and blood glucose;
- Telemedicine can reduce hospital visits for minor conditions.

### What this study adds

- The study gives evidence that telemedicine in contagious pandemic situations is effective in triaging and sometimes diagnosing and providing complete treatment to women in need of Obstetrics and Gynaecology services;
- Telemedicine services in a pandemic have high satisfaction scores;
- It also gives evidence of a lesser proportion of patients who remain with compromised care as they cannot complete their investigations using telemedicine during a contagious pandemic.

## Competing interests

The authors declare no competing interests.
